# Anti NMDA receptor encephalitis manifested as acute psychosis- case report

**DOI:** 10.1192/j.eurpsy.2025.2224

**Published:** 2025-08-26

**Authors:** M. Kasjaniuk, M. Urban-Kowalczyk

**Affiliations:** 1 Central Teaching Hospital of the Medical University; 2 Department of Affective and Psychotic Disorders, Medical University, Łódź, Poland

## Abstract

**Introduction:**

The rapid change a variety of the clinical picture in case of autoimmune encephalitis remain a diagnostic challenge.

**Objectives:**

Diagnostics and therapeutic process as of the atypical psychosis.

**Methods:**

A 40-year-old woman, since 2 weeks she expressed catastrophic delusions and dysphoria. Next she revealed tinnitus, psychosensory disturbances, disorganization of thinking and behavior and the first grand mal seizure occurred. In the neurology ward were described psychogenic disorders and she was referred to the psychiatric ward, where catatonic schizophrenia was diagnosed. She was disoriented, with periodic episodes of arousal and freezing, negativism and command automatism. She was hallucinating visually, auditorily, olfactorily, and cenesthetically, expressed delusions of catastrophe, influence, and persecution. Despite modifications of treatment (aripiprazole, haloperidol, olanzapine, quetiapine, clozapine, valproate) she didn’t improve. In the 4^th^ week of hospitalization, an episode of fever of unknown etiology occurred and she was transferred to a higher referral psychiatric ward. Next fluctuating disturbances of consciousness, myoclonus, bilateral palmomental and Babinski’s sign and swallowing disorders were observed. Due to the lack of therapeutic effect tiapride was administer with slight improvement.

**Results:**

Laboratory tests: CRP, VDRL, anti-HIV antibodies - absent, ceruloplasmin, ANA, anti-TPO, anti-TSHR antibodies, tumor markers – normal range.

No changes in contrast-enhanced MRI head. In EEG moderate-grade, extensive changes in the fronto-central and frontotemporal leads bilateral.

CSF examination – normal range, negative result for 14-3–3 protein

In a panel for antibodies typical of autoimmune encephalitis - presence of anti-NMDA antibodies. Then autoimmune encephalitis was diagnosed. She was initially treated with immunoglobulins, with slight effects. Subsequently, effective immunosuppressive treatment with mycophenolate mofetil and methylprednisolone was administered. Further, the psychosis resolved, with the normalization of the neurological signs. The patient’s in stable mental and somatic condition was discharged without further psychopharmacology.

**Image 1:**

**Figure:**
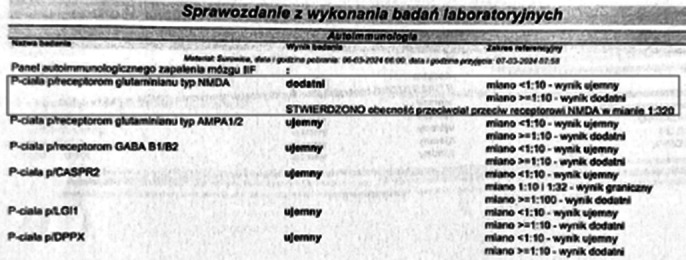

**Image 2:**

**Figure:**
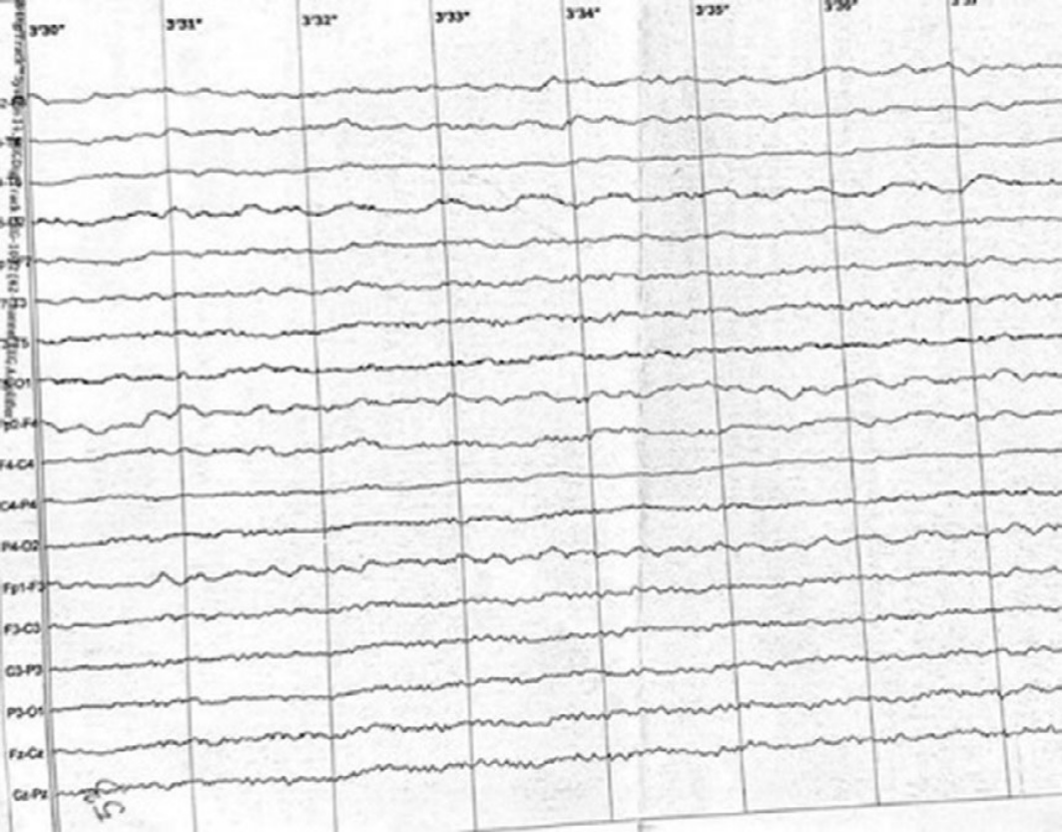

**Conclusions:**

In anti NMDA receptor encephalitis affective symptoms, psychosis, catatonia, consciousness disorders, as well as neurological symptoms occurred. In majority of cases no changes are observed in brain imaging. EEG examination shows non-specific changes in 90% of cases. About 80% of patients recover without functional impairment, but in 7% disease is lethal. Crucial is to determine the etiology of atypical symptoms in order to implement adequate therapy as soon as possible.

**Disclosure of Interest:**

None Declared

